# New Strategies for the Next Generation of Matrix-Metalloproteinase Inhibitors: Selectively Targeting Membrane-Anchored MMPs with Therapeutic Antibodies

**DOI:** 10.1155/2011/191670

**Published:** 2010-10-28

**Authors:** Laetitia Devy, Daniel T. Dransfield

**Affiliations:** ^1^Merck Serono S.A., 9 Chemin des Mines, Case postale 54, 1211 Geneva 20, Switzerland; ^2^Cell Biology and Translational Research, Dyax Corp., 300 Technology Square, Cambridge, MA 02139, USA

## Abstract

MMP intervention strategies have met with limited clinical success due to severe toxicities. In particular, treatment with broad-spectrum MMP-inhibitors (MMPIs) caused musculoskeletal pain and inflammation. Selectivity may be essential for realizing the clinical potential of MMPIs. Here we review discoveries pinpointing membrane-bound MMPs as mediators of mechanisms underlying cancer and inflammation and as possible therapeutic targets for prevention/treatment of these diseases. We discuss strategies to target these therapeutic proteases using highly selective inhibitory agents (i.e., human blocking antibodies) against individual membrane-bound MMPs.

## 1. Introduction

Matrix metalloproteinases (MMPs) are zinc-dependent endopeptidases belonging to a larger family of proteases known as the metzincin superfamily. MMPs play an important role in tumor progression and invasion of inflammatory cells by degrading the extracellular matrix (ECM). Among all MMPs, six (MMP-14, -15, -16, -17, -24, and -25) are referred to as membrane anchored-MMPs (MT-MMPs) [1]. MMP-23 known as CA-MMP (Cysteine array matrix metalloproteinase) is also a membrane-bound MMP but is anchored to the membrane via an N-terminal signal peptide and is structurally distinct from all other MMPs [2, 3]. This paper will outline the new strategies to select highly selective drugs using monoclonal antibodies. A special emphasis will be put on the properties of membrane-bound MMPs and the scientific basis which makes pursuing them attractive as therapeutic targets in cancer and inflammation.

## 2. MMP-Inhibitors in the Clinic

Except AZD1236 which is currently being developed by AstraZeneca for potential treatment of chronic obstructive pulmonary disease (COPD) and CTS-1027 from Conatus Pharmaceuticals currently being evaluated in a phase II clinical trial in combination with pegylated interferon (Pegasys) and ribavirin (Copegus) in refractory hepatitis C patients, there are currently no synthetic or biologic MMPIs in clinical trials for cancer or arthritis. This is mostly due to the failure of early studies with compounds containing zinc-chelating groups, such as hydroxamates [4–6]. A tetracycline derivative, doxycycline, in subantimicrobial doses (Periostat; CollaGenex Pharmaceuticals Inc., Newtown, PA, USA) is currently the only MMPI approved by the US FDA and is used as an adjunct therapy in adult periodontitis [7]. The use of tetracyclines for the treatment of arthritic diseases is limited, although doxycycline has been shown to improve some disease parameters as well as reducing the levels of collagenase activity in some patients with rheumatoid arthritis (RA) [8, 9]. Topical doxycycline is also used to enhance healing of chronic wounds [10].

## 3. Drawbacks of Broad-Spectrum MMP-Inhibitors

Numerous studies in different preclinical cancer models demonstrate the ability of hydroxamate-based MMPIs to delay primary tumor growth and block metastasis [11–13]. Unfortunately, these MMP intervention strategies have met with limited clinical success and severe toxicities [1, 14, 15]. Most of the MMPIs eventually demonstrated side effects after short-term dosing (e.g., marimastat) or prolonged treatment (e.g., BMS-275291) related to musculoskeletal pain and inflammation [16, 17]. The mechanism of these toxicities is widely assumed to be due to the poor selectivity of these compounds [18] but this has not been confirmed. In addition, it is now recognized that among MMPs, some possess cancer-promoting activities while others tumor-inhibiting functions [19] underlining the risk of using broad-spectrum MMPIs. Along these lines, *in vivo* studies have demonstrated that broad-spectrum MMPIs promote metastasis of breast carcinomas as well as lymphomas to the liver in mice [20, 21]. The upregulation of proangiogenic factors observed in the livers of mice treated with such inhibitors supported a direct effect on the angiogenic process [22]. Alternatively, the broad spectrum MMPIs might also inhibit proteases whose activity generates angiostatic factors. A pyrimidine-2,4,6-trione derivative, belonging to the class of orally-available selective MMPI for MMP-2, -9, and -14 was not associated with the occurrence of adverse side effects that might reduce the therapeutic potential of these drugs [23] demonstrating the importance of drug selectivity.

## 4. Antibody-Based Therapeutic Agents

**Figure 1:**
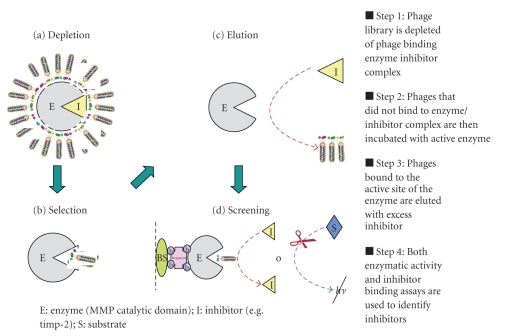
MMP inhibitor selection strategy.

Successful therapeutic intervention may critically depend on potently inhibiting one or more MMPs that contribute to disease progression while not inhibiting related MMPs that may be beneficial to the host or if inhibited lead to clinical toxicities. For example, increased expression of MMP-12 by colon carcinoma cells is associated with increased survival [24], and MMP-8 deficient male mice display increased skin cancer susceptibility [25] due to an increased inflammation which delays wound healing [26]. Antibody-based biotherapeutic agents (e.g., human antibodies from phage display libraries) may fulfill this need as they may offer the desired selectivity and potency required for disease-modifying activity [27]. The high affinity binding of a monoclonal antibody to its target confers the potential for high potency and selectivity coupled to a drug scaffold with excellent pharmacological properties. Combining our human antibody phage display library with automated selection and screening strategies (Figure 1) [28], we have identified highly selective antibody-based MMP inhibitor of MMP-14 (DX-2400). DX-2400 displays antih-invasive, antitumor, and antiangiogenic properties and blocks proMMP-2 processing [29]. HT-1080 cells, which express MMP-14 and MMP-2, were used to assess the effect on MMP-2 activity by the selective inhibition of endogenous MMP-14 by DX-2400. DX-2400 blocked proMMP-2 processing, whereas a polyclonal rabbit anti–MMP-14 antibody, which does not inhibit MMP-14 activity, failed to inhibit proMMP-2 activation. DX-2400 inhibited HUVEC tube formation (IC_50_~ 6 nmol/L) and inhibited migration of HUVECs in a fibrin gel bead assay whereas proliferation was unaffected. DX-2400 also inhibited VEGF165-induced invasion of HUVECs. Our *in vivo* studies demonstrated that DX-2400 markedly affected tumor growth of human breast cancer (MDA-MB-231) xenograft tumors when used as a single agent or in combination with bevacizumab. Combination therapy with antiangiogenic and novel antiproteolytic agents such as DX-2400 represents a promising approach that may produce a synergistic antitumor effect and a survival benefit for patients. In the MDA-MB-231 model, the antitumor effect of DX-2400 was associated with a strong decrease in tumor vascularization. DX-2400 treatment also induced a significant reduction of MMP activity, supporting an antiproteolytic effect of this antibody. DX-2400 showed *in vivo* activity at all dosing schedules tested, with every other day treatment regimen yielding the highest efficacy. DX-2400 showed activity against the HER2–positive BT-474 xenografts when used as a single agent or in combination with paclitaxel. These results make DX-2400 an attractive candidate for breast cancer patients, especially in cases where hormonal therapy and/or therapy with Herceptin (trastuzumab) is not effective. DX-2400 combined with bevacizumab resulted in increased tumor growth delay *in vivo*. DX-2400 did not alter the growth of MCF-7 (MMP-14 negative) derived tumors, showing MMP-14 dependency for DX-2400 action. In addition to its effects on primary tumor growth, DX-2400 also significantly reduced the number of metastatic foci in the MDA-MB-231 orthotopic model and in the i.v. mouse B16F1 melanoma model. Our findings pharmacologically validate the role of MMP-14 in oncology and emphasize the therapeutic potential of specific antibody-based MMP inhibitors such as DX-2400 for the treatment of solid tumors.

Extending our approach to human blocking antibodies targeted against other MMPs will allow for a clear delineation of their role in various pathophysiological diseases and potentially serve as therapeutic agents in cancer and inflammatory diseases.

## 5. Properties of Membrane-Anchored MMP: Structure, Regulation, and Tissue Localization

**Figure 2:**
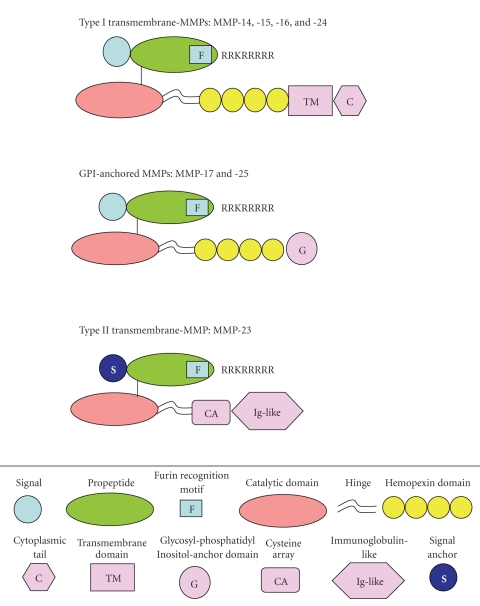
Primary structure of membrane-anchored MMPs.

The primary structure of membrane-anchored MMPs consists of all the domains characteristic of other MMPs (Figure 2), except for MMP-23 which does not contain the hemopexin domains [2]. In addition, MMP-14, -15, -16, and -24 are type I transmembrane proteins [30] with a short cytoplasmic tail at the C-terminus [31]. MMP-17 and -25 are glycosylphosphatidylinositol- (GPI-) anchored to the cell surface and have a very short cytoplasmic tail which is removed in the endoplasmic reticulum during incorporation of the GPI anchor [32]. Common to all membrane-bound MMPs is the 11 amino acid insertion with a conserved RRKRRRRR sequence, representing a furin cleavage site, located at the end of the propeptide domain. With the exception of MMP-17 and -25, the membrane-associated MMPs also have an insertion of 8 amino acid residues within the catalytic domain or membrane-type- (MT-) loop [33]. MMP-23 has a unique cysteine-rich, proline-rich and interleukin (IL-1) receptor type II-like domains.

L_(20)_GAALSGLCLLSALALL_(36)_ is required for this unique membrane localization as a signal anchor [2]. The C-terminal domain of MMP-23 is considerably shorter than other MMPs and shows no sequence similarity to hemopexin [3].

Each member of the membrane-bound MMP subfamily maps to a distinct chromosome which indicates that chromosomal transposition events have played a major role in the evolutionary diversification of this gene family [61]. MMP-14 and MMP-15 share 82.5% amino acid sequence homology [62], MMP-14 and MMP-16, 66% [63], MMP-14, and MMP-17, 29% and MMP-17 and -25, 77% [3]. MMP-17 and MMP-25 display 39% and 45% amino acid identity to MMP-14 [64], suggesting that GPI- anchored MMPs are structurally and functionally distant from MT-MMPs. Expression of membrane-bound MMPs is differentially controlled at the transcriptional level [63, 65, 66]. The mechanism responsible for membrane-bound MMP activation is mediated intracellularly by furin-like proteases [66]. TIMPs (-1 to -4) inhibit enzyme catalytic activity but also regulate MT-MMP processing and internalization, determining the amount of mature enzyme on the cell surface [40, 67, 68]. GPI-anchored MMPs can be released from the cells in exosomes and transferred to other cell types in a paracrine manner where they can elicit biological effects [57]. Like secreted MMPs, membrane-bound MMPs can cleave extracellular matrix (ECM) molecules, as well as chemokines, cytokines, and growth factors [40] (Table 1). The limited ECM degrading activity of the GPI-anchored MMPs is in accordance with their reported inability to facilitate invasion of basement membranes [69] and invasion of a fibrin gel *in vitro* [70]. MMP-17 and MMP-25 possess the ability to cleave non-ECM proteins [57]. The hemopexin-like domain of MMP-14 and -16 are essential for the cleavage of fibrillar collagens. In addition, membrane-bound MMPs are known to cleave and activate secreted MMPs, first described for the activation of MMP-2 by MMP-14 through interaction with TIMP-2 [30, 71]. MMP-14 also has been shown to activate proMMP-13 [72]. MMP-14 and -15 mRNA transcripts are expressed in a number of tissues but are distributed quite differently. MMP-16, -17, -23, -24, and -25 have a more restricted pattern of expression (Table 2).

## 6. Membrane-Anchored MMPs in Cancer

The process of cancer progression involves the action of multiple proteolytic systems, in which membrane-anchored MMPs play a pivotal role. Their localization at the focal cell surface results in conditions especially suitable for cancer cells to progress and invade the ECM [57]. Membrane-bound MMPs are expressed not only by cancer cells but also by the surrounding tumor stromal cells. They also play a critical role in the development of the desmoplastic reaction characteristic of cancer tissues such as breast, pancreatic, and lung. Changes in the tumor microenvironment due to the desmoplastic reaction may benefit the tumor by enhancing proliferation, inducing a more invasive malignant phenotype, and increasing chemoresistance. 

### 6.1. Type I Transmembrane MMPs

Extensive work from the Weiss laboratory demonstrated that select type I membrane-anchored MMPs (MMP-14 and MMP-15) function as direct-acting, proinvasive factors for expansive growth of primary tumors within a tridimensional collagen type I matrix. The proinvasive, angiogenic, and metastatic activities of MMP-14 and MMP-15 are unique relative to all other MMP family members and cannot be mimicked *in vivo* by secreted MMPs, MMP-1, -2, -3, -7, -9, or -13 [87]. MMP-14 drives invasion by functioning as a pericellular collagenase [88], an activator of proMMP-2 [30, 89], and is directly linked to tumorigenesis and metastasis [90–92]. MMP-14 expression is elevated in various human carcinomas including uterine cervix [93], stomach [94], lung [95–97], breast [98], colon [99], head and neck [100], malignant brain tumors [101], and melanoma [102]. MMP-14 immunostaining in primary tumor specimens is a prognostic predictor in patients with medullary thyroid carcinomas [103] or carcinoma of the larynx [104]. High MMP-14 expression is associated with early death of patients with supraglottic carcinoma [105], colorectal carcinoma [106], or breast cancer [107] and is correlated with lymph node metastases, progression, invasion, poor clinical stage, larger tumor size, and with increasing tumor stage [108, 109]. The expression of MMP-14 and MMP-2 correlates with the depth of tumor and vascular invasion in human colon cancer [110].

MMP-15 also plays a key role in cancer progression, tumor invasion, and metastasis [111]. MMP-15 mRNA is expressed in breast carcinoma [112] and pancreatic cancer tissues [113]. Higher levels of MMP-15 are observed in nonsmall cell lung carcinomas (NSCLCs) relative to squamous cell carcinoma (SCCs) and normal lung tissues which indicate that MMP-15 may be a viable molecular diagnostic marker for NSCLCs [96]. Chemokine CXCL12 upregulates MMP-15 expression in glioma cells and serves as an effector of CXCR4 signaling in these cells by promoting cell invasion [46]. MMP-15 has antiapoptotic activity [114] and may connect metastasis and resistance to cell death by apoptosis through an unknown regulatory mechanism.

MMP-16 is expressed in human hepatocellular carcinoma and correlates significantly with capsular invasion [110]. MMP-16 is expressed by and promotes invasion of melanoma cells [115].

MMP-24 mRNA is highly expressed in brain tumors, including astrocytomas and glioblastomas [53]. MMP-24 gene silencing by RNAi can suppress the invasiveness of SKOV-3 ovarian cancer cells *in vitro*, which may provide a new therapeutic approach for this type of cancer [116].

### 6.2. GPI-Anchored MMPs

GPI-anchored MMPs are associated with progression of human cancer by mechanisms different from the type I transmembrane MMPs. There is an excellent review on the properties and expression of MMP-17 and MMP-25 in cancer published by Sohail and coworkers [57]. Data suggest that GPI-anchored MMPs do not act as progelatinase activators, are mostly non-ECM degrading enzymes, and do not promote cell migration and invasion. MMP-17 promotes primary tumor growth and lung metastasis in preclinical models. MMP-17 is strongly expressed in human breast cancer cells and in metastatic cells in human lymph nodes [117]. Chabottaux et al. applied experimental and spontaneous models of lung metastasis using human breast adenocarcinoma MDA-MB-231 cells overexpressing or not MMP-17 and found that MMP-17 promotes lung metastasis by disturbing the tumor vessel integrity and thereby facilitating tumor cell intravasation [118]. Human MMP-25 is expressed by leukocytes and neutrophils, and in colon, urothelial, brain, and prostate cancers [59, 119–122]. MMP-25 was suggested to contribute to disease progression in gliomas [123]. Expression of MMP-25 in HCT-116 human colon cancer cells promotes tumorigenesis in nude mice. Histologically, the MMP-25-expressing tumors demonstrate an infiltrative leading edge. Strong MMP-25 staining was detected in inflammatory-like cells consistent with the known expression of MMP-25 in leukocytes [64].

### 6.3. Type II Transmembrane MMP

The body of work on MMP-23 in cancer is still very limited when compared to other MMPs. It is interesting to note that the presence of MMP-23 in MDA-MB-231 cells and its involvement in cell invasiveness after gene silencing by RNAi have been reported [124].

## 7. Membrane-Anchored MMPs in Inflammatory Diseases

Inflammatory disease encompasses a huge array of disorders that can be very localized, regional, or systemic. MMPs act on proinflammatory cytokines, chemokines, and other proteins to regulate varied aspects of inflammation and immunity. Numerous targets of MMP activity that directly affect components of the immune system inflammatory pathways have been described in a review by Cauwe et al. [125]. 

### 7.1. Type 1-Transmembrane MT-MMPs

Uncontrolled cell migration and tissue invasion are one of the important factors that promote progression of diseases such as RA. In RA, inflamed synovial pannus tissue invades into cartilage and bone, resulting in dysfunctioning joint. Recent results from Itoh's group based on Western blot analysis of primary synovial cells and immunohistochemical analysis of RA joint specimens have highlighted the key role played by MMP-14 in the progression of RA by promoting cartilage invasion by synovial pannus tissue [126]. Jain et al. also showed that invasive potential of human rheumatoid tenosynovial cells is partly MMP-14 dependent [127]. MMP-15, which activates proMMP-2 and proMMP-13 and is involved in TNF*α* processing (Table 1), also may facilitate inflammatory tissue destruction in RA [128].

Johnson et al. have highlighted MMP-14 as a potential target for the stabilization of atherosclerotic lesions [129]. Furthermore, they also published a study on the effect of a broad spectrum nonselective MMPI in this mouse model in which it was demonstrated that the nonselective MMPI has no beneficial effects on atherosclerosis [130]. Subsequently using double-deficient mice, they observed that with regards to atherosclerotic plaque disruption, some MMPs are beneficial and some are detrimental [131]. MMP-24 plays a role in the pathogenesis of renal tubular atrophy and end-stage renal disease [132]. MMP-24-null mice do not develop neuropathic pain induced by peripheral nerve lesions [133].

### 7.2. GPI-Anchored MMPs

MMP-17 is involved in cartilage destruction by activating ADAMTS-4 [134, 135]. Contrary to the reported role of MMP-17 as a TNF*α* sheddase [58], the lipopolysaccharide- (LPS-) induced release of TNF*α* from mmp-17(−/−) macrophages was similar to that in wild-type cells [136]. Using quantitative RT-PCR, Bar-Or and colleagues have systematically analyzed the expression of MMP members in subsets of leukocytes isolated from the blood of normal individuals [76]. MMP-17 is significantly expressed in B cells. A recent study from Shiryaev and colleagues highlights the key role played by MMP-25 in the proteolytic pathway in multiple sclerosis (MS) [137]. MMP-25 is superior to MMP-2, -8, -9, -10, -12, -14, -15, -16, -17, and -24 in cleaving myelin basic protein (MBP) isoforms. Proteolysis of the Golli-MBP isoforms by MMP-25 followed by the stimulation of the specific autoimmune T cells causes increased inflammation. This leads to the further upregulation of the activity of multiple MMPs and the massive cleavage of MBP in the brain resulting in demyelination and MS [137]. MMP-25 is a novel and promising drug target in MS especially when compared with other individual MMPs.

### 7.3. Type II Transmembrane MMP

MMP-23 mRNA is expressed in chondrocytes and osteoblasts, suggesting a role in some aspect of cartilage or bone formation [138]. ADAM-12 and MMP-23 are coexpressed in painful tendinopathy [139] suggesting a role for these in this inflammatory disorder.

## Figures and Tables

**Table 1:** Substrates for membrane-bound MMPs.

	Gene/Name	*M*_*r*_ latent	*M*_*r*_ active	Substrates
	MMP-14 (MT1-MMP)	66,000	56,000	Collagen I, II, III [34, 35], and IV [36–38], Gelatin, cartilage aggrecan, perlecan, fibronectin, vitronectin, nidogen, laminin, pro-TNF*α* [34, 35]; proMMP-2 [39]; proMMP-13 [40]; Galectin-3; MCP-3 [41]; SDF [42]; cell surface CD44 [43]; tTG [44].
Type I transmembrane MMPs	MMP-15 (MT2-MMP)	72,000	60,000	Gelatin; fibronectin; tenascin; nidogen; aggrecan; perlecan and laminin [35]; tTG [44]; proTNF*α* [35]; LRP (CD91) [45]; CXCL12 [46]; proMMP-2 [47].
	MMP-16 (MT3-MMP)	64,000	52,000	Native Collagen III, *α*2(I) collagen chain; cartilage proteoglycan; gelatin; casein; fibronectin; vitronectin; laminin-1; transferring; *α*1-proteinase inhibitor and *α*2-macroglobulin [48, 49]; tTG [44]; proMMP-2 [50].
	MMP-24 (MT5-MMP)	63,000	45,000	Fibronectin; Proteoglycans and cadherins [51, 52]; Gelatin; proMMP-2 and -9 [53, 54]; KISS-1 [55].

GPI-anchored MMPs	MMP-17 (MT4-MMP)	57,000	53,000	Gelatin [56]; alpha2-macroglobulin; ADAMTS-4; low density lipoprotein receptor related protein [57]; Fibrin/Fibrinogen; pro-TNF-alpha cleaved by mouse MMP-17 [58].
	MMP-25 (MT6-MMP)		56,000	Collagen IV; Gelatin; Fibrin/Fibrinogen [59, 60]; Fibronectin; laminin-1, alpha2-macroglobulin; ADAMTS-4; Chondroitin and dermatan sulfate proteoglycan; alpha1 proteinase inhibitor; urokinase plasminogen activator receptor, Galectin-3 [57].

Type II transmembrane MMPs	MMP-23	43,900	?	Unknown.

*M*_*r*_: relative molecular mass.

**Table 2:** Expression of membrane-bound MMPs in normal tissues.

	Gene/name	cDNA	Expression in normal tissues
	MMP-14 (MT1-MMP)	Isolated from a human placenta cDNA library [73]	Highly expressed in ossifying tissues and during mouse embryogenesis, where it is coexpressed with MMP-2 [74]. Low expression in normal conditions.
Type I transmembrane MMPs	MMP-15 (MT2-MMP)	Isolated from a mouse lung cDNA library [75]	Highly expressed in T cells [76], rat smooth muscle cells [77], and endothelial cells [78]. Expressed in hepatocytes and biliary epithelial cells [79], in cytotrophoblasts [80], in activated NK-cells [81], and in microglial cells [82].
	MMP-16 (MT3-MMP)	Isolated from placenta cDNA library [63]	Expressed in human brain tissues (microglial cells) [62]; T cells [76]; endothelial cells [78, 83]; Langerhans cells following cell activation [84].
	MMP-24 (MT5-MMP)	Isolated from mouse brain cDNA library [54]	Predominantly expressed in the central nervous system [85] and in T lymphocytes [76].

GPI-anchored MMPs	MMP-17 (MT4-MMP)	Isolated from a human breast carcinoma cDNA library [66]	Expressed in monocytic cells [86], leukocytes, brain, ovary, testis, and colon [66].MMP-17 mRNA is significantly represented in B cells [76].
	MMP-25 (MT6-MMP)	Isolated from peripheral blood leukocytes [64]	Predominantly expressed in leukocytes [64]. In rats, mRNA peak expression levels in testis, kidney, and skeletal muscle [85].

Type II transmembrane MMPs	MMP-23	Isolated from an ovarian cDNA library [3]	Abundantly expressed in normal tissues in adults under quiescent conditions and predominantly expressed in reproductive tissues and others such as heart [3].
